# Inferior retinotomy and silicone oil tamponade for recurrent inferior retinal detachment and grade C PVR in eyes previously treated with pars plana vitrectomy or scleral buckle

**DOI:** 10.1186/s12886-015-0167-z

**Published:** 2015-12-09

**Authors:** Raffaele Mancino, Francesco Aiello, Elena Ciuffoletti, Emiliano Di Carlo, Angelica Cerulli, Carlo Nucci

**Affiliations:** Ophthalmology Unit, Department of Experimental Medicine and Surgery, University of Rome “Tor Vergata”, Via Montpelier 1, 00133 Rome, Italy

**Keywords:** Recurrent retinal detachment, Scleral buckle, Pars plana vitrectomy, Inferior retinotomy, Silicone oil tamponade

## Abstract

**Background:**

One of the most challenging problems in vitro-retinal surgery is the recurrence of retinal detachment in the context of high-grade proliferative vitreoretinopathy (PVR). The aim of our retrospective study was to assess the surgical outcomes of pars plana vitrectomy, 180° inferior retinotomy and silicone oil tamponade combined with phacoemulsification and IOL implantation for recurrent inferior retinal detachment with grade C PVR in phakic eyes. The study was carried out at tertiary referral centre - University Hospital of Rome “Tor Vergata”.

**Methods:**

Retrospective analysis of 33 eyes affected by recurrent inferior retinal detachment and grade C PVR after primary encircle scleral buckling (SB group – 12 eyes), or pars plana vitrectomy (PPV group – 21 eyes). All patients subsequently underwent PPV and silicone oil tamponade at our Institution. The first outcome measure was retinal reattachment, and second outcomes were reoperation rates, best-corrected visual acuity (BCVA) and postoperative complications.

**Results:**

All patients in the SB group and 19 (90 %) patients of the PPV group achieved retinal reattachment. Final BCVA was better in the SB group (*p* = 0.045). Two eyes in the PPV group required a third vitrectomy with heavy silicone oil tamponade. Postoperative complications included silicone oil in a deep anterior chamber (3 eyes in each group), untreatable hypotony in 1 eye in the PPV group (that led to enucleation due to phthisis bulbi), and elevated intraocular pressure in 3 patients (2 eyes in the PPV group).

**Conclusions:**

Phacoemulsification with IOL implant, PPV with silicone oil tamponade associated with 180° inferior retinotomy may lead to better anatomical success in patients who have previously undergone SB procedure for inferior retinal detachment repair compared with eyes that underwent a primary PPV.

## Background

In 1979, Machemer described how to perform a therapeutic cut of the retina, achieving retinotomy during pars plana vitrectomy (PPV) [1]. He also defined indications for retinotomy, particularly when proliferative vitreoretinopathy (PVR) is responsible for circumferential/axial retinal traction [2]. Over the years, these procedures have been combined in many studies [3–11] culminating in the recent multicentre Silicone Oil Study [12–15]. Blumenkranz et al. [13] suggested that retinotomy is essential in eyes which have undergone primary PPV for retinal detachment as they frequently develop post-operative PVR [3, 6, 7]. One of the most challenging surgical problems is the inferior retinal detachment (RD), due to the absence of an optimal intravitreal tamponade agent. This is due to the inadequate surface tension and consequent reduced tamponade effect on the inferior part of the retina. In light of this difficulty, PVR can further complicate an inferior re-detachment after PPV. Furthermore, the silicone oil tamponade left in the eye during PPV for inferior retinal detachment may lead to the sequestration of retinal epithelium pigment cells over the inferior retina resulting in new PVR [16, 17]. Alternatively a scleral buckle (SB) can in theory offer good support to the retina, however this technique remains technically demanding with an increase in potential intraoperative and postoperative complications [18–20]. The purpose of this study was to investigate surgical and visual outcomes of phacoemulsification with intraocular lens (IOL) implant, PPV with inferior retinotomy extended to 180 degrees, and a silicone oil implant to treat recurrent inferior retinal detachment in those previously treated with PPV compared to SB surgery. The primary outcome was anatomical surgical success, defined as a complete retinal re-attachment at last follow up. Secondary outcomes included visual acuity (VA), VA improvement, the number of subsequent operations required for reattachment and post-operative complications.

## Methods

The Institutional Review Board of the “Tor Vergata” University Hospital approved this audit. The study has been performed in accordance with the ethical standards laid down in the 1964 Declaration of Helsinki and its later amendments. Data was obtained by a retrospective study of the medical records of eyes affected by inferior retinal redetachment subsequent to primary PPV or SB surgery performed for inferior rhegmatogenous RD. Inclusion criteria included the presence of grade C PVR according the Machemer’s classification [21], defined as the presence of full thickness rigid retinal folds in one area with heavily condensate vitreous organization. We identified 33 eyes of 33 consecutive patients that underwent primary retinal repair at our Centre or were referred to our Institution for further management after primary RD surgery had failed. All patients underwent the second surgical procedure at our Institution by a single surgeon (RM) between 2008 and 2012. Patients were divided into two groups according to the first RD surgery repair technique; the first group had undergone previous SB surgery (12 eyes of 12 patients), whilst the second group had undergone pars plana vitrectomy (PPV) (21 eyes of 21 patients). All patients in the SB group had received encircling band and circumferential buckle placed at the time of the primary RD repair.

Surgery was performed after informed consent was obtained from all patients. Exclusion criteria were: proliferative diabetic retinopathy, high myopia, penetrating eye injury, uveitis, more than one previous retinal detachment surgery, or with <12 months postoperative follow-up. Eyes that underwent retinotomy less then 180 degrees due to a less severe retinal detachment status were excluded from this study.

Each patient underwent pre- and post-operative complete ophthalmic examination, which included logarithm of the minimum angle of resolution (logMAR), best-corrected visual acuity (BCVA), applanation tonometry, slit lamp biomicroscopy and dilated binocular indirect ophthalmoscopy with scleral depression to evaluate retinal status, and PVR.

Outcome variables included BCVA, retinal reattachment rate at final follow-up, the number of cases requiring reoperation, and postoperative complications. Data were collected at follow-up examination on postoperative day 1, 1 week, 1 month, 3 months, 6 months, 1 year and 2 years post-surgery. The mean time between first surgery and retinal redetachment diagnosis was 14 days (range: 3 to 30 days). BCVA was evaluated separately for each eye with a standard ten-letter logarithmic chart 4 m [22], ^28^. Values of 1.3 logMAR (equivalent to 20/400 Snellen), 1.6 logMAR (20/800) and 2.0 logMAR (20/2000) were assigned when the first line of the chart was read at 2 m, 1.1 m and 40 cm respectively; hand motion at 40 cm was classified as 3.0 logMAR (equivalent of 20/20,000) [23]. Visual loss was classified according to the World Health Organization (WHO) criteria [24]: low vision = BCVA >0.5 to 1.3 logMAR (<20/70 to 20/400 Snellen) (ICD-10 categories 1–2); blindness = BCVA >1.3 logMAR (<20/400) (ICD-10 categories 3–5).

### Surgery

All patients were phakic at the time of re-detachment surgery repair. All eyes that had undergone PPV were tamponaded with oil during the first surgery. Observing that all re-detachments occurred between 3 and 30 days after the first surgery, all eyes had silicon oil in the vitreous cavity at the time of secondary re-detachment surgery. Thus, standard 3.0 mm clear corneal incision phacoemulsification surgery and implantation of a three-piece IOL was performed in all eyes to allow an adequate vitreous base shaving, and anterior PVR dissection - as described by Quiram et al. [25] A 23-gauge pars plana vitrectomy was then performed (with silicon oil removal for eyes that had received a previous PPV). A 360-degree scleral depression was then performed to allow complete anterior vitreous base visualization and removal. Perfluorocarbon was used to protect the macula from haemorrhages and to flatten the retina during the procedure [26]. PVR membranes were peeled from the retinal surface using an illuminated pick, retinal spatula or forceps. Full thickness endocautery was used to delimit the inferior 180-degrees of the retina before performing vitrector-assisted relaxing retinotomy. Subsequent posterior retinal turnover was performed to remove subretinal membranes if present, with direct visualization. The retina anterior to the retinotomy was completely removed with the high-speed vitrector to prevent neovascularization due to ischemic stimuli [27]. After flattening the retina with perfluorocarbon liquid. a double concentric pattern of endolaser was applied to the retinotomy edges, followed by direct perfluorocarbon liquid/silicone oil (1000 centistokes) exchange to prevent further retina mobilization. In all eyes an inferior iridectomy was performed to prevent pupillary block glaucoma that can arise in pseudophakic eyes after vitrectomy and silicone oil injection as previously described [28, 29]. No position restrictions were given postoperatively.

### Statistics

Mean, standard deviation (SD), median, 25 % and 75 % quartiles, minimum and maximum values were computed for all variables. For continuous variables, data was compared using the Student’s t-test or paired t-test. For categorical variables, comparison between groups was performed using the Chi-square or Fisher exact tests. Significance was set at *p* < 0.05.

## Results

Thirty-three eyes of 33 consecutive patients underwent vitreoretinal surgery: 20 males (60.6 %) and 13 females (39.3 %) with recurrent inferior RD and grade C PVR. The mean age was 56.2 ± 12.3 (±SD) years (range: 30 to 71 years). Thirty-one (93.9 %) patients were Caucasian, 1 (3 %) African and 1 (3 %) Asian. All patients were admitted to the Department of Vitreoretinal Surgery of the University of Rome “Tor Vergata” between January 2008 and June 2012 for secondary RD surgery repair. The mean follow-up period was 42 months (range: 12 to 60 months). Baseline characteristics of the two groups were homogeneous with no significant statistical difference between groups in terms of age, gender, PVR stage, initial BCVA and macular status (Tables 1 and 2). All patients were phakic. At the time of surgery, 12 patients (36.4 %) had previously undergone encircling scleral buckling (SB group) and 21 (63.6 %) had undergone pars plana vitrectomy (PPV group) (Table 2). Inferior retinal redetachment occurred between 3 and 30 days after the first operation (mean 14 days). In the PPV group all underwent 23-gauge pars plana vitrectomy as the primary and secondary surgery. None of the patients had a concomitant macular hole. At the end of surgery it was reported the retina was attached in all cases.

**Table 1:** Characteristics of all participants in the study

Code	First surgical technique	Age (yrs)	Sex	PVR grade	Initial BCVA (logMAR)	Final BCVA (logMAR)	Macular status
01	SB	49	M	C2	3.0	0.7	Detached
02	SB	43	F	C1	0.5	0.2	Attached
03	PPV	30	F	C2	3.0	1.3	Detached
04	PPV	45	F	C1	1.0	1.0	Detached
05	PPV	49	M	C2	0.7	1.8	Detached
06	PPV	61	M	C2	0.7	0.7	Attached
07	SB	51	F	C1	0.4	0.7	Attached
08	PPV	63	F	C1	0.3	0.5	Attached
09	PPV	69	M	C2	0.2	0.1	Attached
10	SB	59	M	C1	0.7	0.6	Attached
11	PPV	70	M	C1	0.3	0.7	Attached
12	PPV	31	M	C1	0.5	0.9	Attached
13	PPV	60	F	C1	2.0	1.0	Detached
14	SB	60	F	C2	1.8	0.2	Detached
15	SB	62	M	C2	1.3	0.8	Detached
16	PPV	70	M	C2	1.6	1.3	Detached
17	PPV	40	F	C1	1.8	0.1	Detached
17	SB	38	M	C2	1.6	0.9	Detached
19	PPV	45	M	C1	1.4	0.6	Detached
20	PPV	69	F	C2	0.3	0.2	Attached
21	PPV	71	M	C2	0.7	1.8	Detached
22	SB	44	M	C1	0.3	0.5	Attached
23	SB	46	F	C2	1.0	0.7	Attached
24	PPV	50	F	C2	3.0	0.1	Detached
25	SB	57	M	C2	0.2	0.1	Attached
26	SB	67	M	C1	0.4	0.4	Attached
27	PPV	59	M	C1	3.0	0.2	Detached
28	PPV	66	F	C2	0.9	0.7	Attached
29	PPV	70	M	C1	1.6	1.3	Detached
30	SB	70	M	C2	0.6	0.4	Attached
31	PPV	71	M	C1	3.0	1.8	Detached
32	PPV	52	F	C2	1.6	0.9	Detached
33	PPV	69	M	C2	3.0	1.3	Detached

*SB* Scleral Buckling, *PPV* Pars Plana Vitrectomy, *M* Male, *F* Female, *PVR* proliferative vitreoretinopathy, *BCVA* Best-Corrected Visual Acuity

**Table 2:** Characteristics of groups previously treated with scleral buckling (SB) or pars plana vitrectomy (PPV)

Characteristic	SB group (12)	PPV group (21)	P
Age in years (mean ± SD)	53.83 ± 10.15	57.61 ± 13.40	0.404^a^
Sex (No. of male/No. of female)	8/4	12/9	0.719^b^
Grade C PVR (No. of stage 1/No. of stage 2)	5/7	10/11	1.000^b^
Initial BCVA in logMAR (mean ± SD)	0.98 ± 0.82	1.46 ± 1.03	0.177^a^
Final BCVA in logMAR (mean ± SD)	0.51 ± 0.26	0.87 ± 0.56	0.045^a^
Macular status (Attached/Detached)	8/4	7/14	0.083^b^
Retinal reattachment after inferior retinotomy (No. of success/No. of failure)	12/0	19/2	0.523^b^

*SD* standard deviation, *PVR* proliferative vitreoretinopathy, *BCVA* Best-Corrected Visual Acuity

^a^Student t-test

^b^Fisher exact test

### Functional outcome

Preoperative BCVA was 1.28 ± 0.97 logMAR (median 1.00; range 0.2–3.0 logMAR), with significant improvement to 0.74 ± 0.50 logMAR (median 0.70; range 0.1–1.8 logMAR) (Table 3) at final follow up (*p* < 0.003) with all eyes considered together. In particular, the PPV group improved from mean 1.46 ± 1.03 logMar to 0.87 ± 0.56 logMAR (*p* = 0.024). The SB group changed from 0.98 ± 0.082 logMAR to 0.52 ± 0.26 logMAR, reaching only a trend toward significance (*p* = 0.057) (Table 4). Final BCVA was better in the SB group compared with PPV group (*p* = 0.045). All patients underwent standard phacoemulsification cataract surgery and implantation of a three-piece IOL. According to our clinical opinion, no eyes had significant lens opacity despite some patients having undergone previous surgery, in particular those who had undergone PPV.

**Table 3:** Descriptive statistics of all participants’ characteristics

Characteristic	Mean	SD	Median	25 % quartile	75 % quartile	Min	Max
Age (yrs)	56.2	12.2	59	46	69	30	71
Initial BCVA (logMar)	1.28	0.97	1	0.5	1.8	3.0	0.2
Final BCVA (logMar)	0.74	0.50	0.7	0.4	0.1	1.8	0.1

*SD* standard deviation, *BCVA* Best-Corrected Visual Acuity

**Table 4:** Changes of BCVA after inferior retinotomy

Surgical technique	Initial BCVA mean ± SD	Final BCVA mean ± SD	P*
SB	0.98 ± 0.82	0.52 ± 0.26	0.057
PPV	1.46 ± 1.03	0.87 ± 0.56	0.024
SB + PPV	1.28 ± 0.97	0.74 ± 0.50	0.003

*BCVA* Best-Corrected Visual Acuity, *SD* Standard deviation, *SB* Scleral Buckling, *PPV* pars plana vitrectomy

*Assessed with Student paired t-test

### Anatomical outcome

At the last follow up, an attached retina was reported in all patients for the SB group (100 %) and 19 patients of the PPV group (90.4 %). Two patients (9.5 %) of the PPV group needed a third vitrectomy with heavy silicone oil tamponade. No intraoperative complications were observed. Postoperative complications included silicone oil in a deep anterior chamber (3 eyes in each group), untreatable hypotony (1 eye in the PPV group) and elevated intraocular pressure in 3 patients (2 eyes in the PPV group), which was treated successfully in all cases with topical intraocular pressure lowering drugs. Postoperative peripheral argon laser retinal photocoagulation was performed in 7 patients (3 in the PPV group) as intraoperative endolaser treatment was not fully completed. After surgery, the silicone oil was removed in all eyes, with an average interval from the last surgery of 4 months (range: 3 to 6 months). Indications for silicone oil removal were: silicone oil emulsification in the anterior chamber with 2 months of flat retina, or an attached retina for over 3 months. Silicone oil removal was as complete as possible. After silicon oil removal we observed only temporary hypotony in all cases but one in the PPV group, the patient having untreatable hypotony that required enucleation 2 years after the latest surgery due to phthisis bulbi.

## Discussion

Advances in surgical techniques have significantly improved anatomical success rates for rhegmatogenous RD. Thus, current retinal surgery can offer favourable outcomes after RD, with a general success rate of up to 94 % [7, 30]. However, the primary success rate of PPV surgery in patients with a complex RD was shown to fall as low as 74 % [31], despite the fact that PPV for RD has been improved in recent decades by the use of perfluorocarbon liquids, endolaser treatment and wide-angle viewing systems. [25, 32–36] It has been shown that PVR is the most common cause of recurrent RD after surgical repair of rhegmatogenous RD and occurs in 5–11 % of patients [37–40]. Furthermore, it was previously reported that 86 % of eyes with recurrent RD after vitrectomy have a component of anterior PVR [7] and eyes with anterior PVR have poorer surgical prognosis than those with posterior PVR [15, 41, 42]. Consequently, several types of complicated RD can be refractory to treatment and extensive peripheral retinotomy is required [43, 44]. However, due to the high risk of complications and generally poor functional results a relaxing retinotomy is rarely advised as a primary procedure but should be considered when complex PVR is present [35, 45]. In this study we explored anatomical and functional outcomes of 33 phakic eyes with inferior retinal redetachment and grade C PVR, with previous PPV or SB as primary treatment for inferior RD. All patients underwent PPV with 180° inferior retinotomy and silicone oil tamponade (1000 centistokes) combined with phacoemulsification and IOL implantation with good anatomical results. In particular, the SB group reached 100 % retinal reattachment vs 90.4 % in the PPV group. In our cohort all eyes underwent a phacoemulsification with IOL to ensure radical anterior vitreous base dissection to directly release anterior vitreous traction, and reduce vitreous scaffolding for potential proliferative tissue [25]. Moreover an extensive inferior retinotomy was required due to the recurrent inferior detachment along with the presence of high grade PVR and retina folds. In our opinion, having a large circumferential retinotomy is beneficial in cases of recurrent inferior RD where vitreous remnants and the presence of high grade PVR increases the probability of having multiple small retinal breaks (sometimes undetected). We speculate that, with high grade PVR, the coexistence of multiple tiny close retinal breaks is possible. In this scenario, the tractive forces responsible for these retinal holes are not localized in a single point but spread throughout a diffuse area. We believe that in this case we should avoid treating these as multiple single tears, and perform a relaxing retinotomy (obtaining a single larger tear with a continuous linear shape) to reduce all traction forces due to the PVR. In our cohort, retinotomies were 180 degrees wide, reflecting not only the severity of the retinal disease but also to obtain a good silicon oil tamponade of the ends of the retinotomy when the patient was in the upright position [46]. Although in our groups the difference in reattachment rate is not statistically significant, we speculate that the better anatomical results in the SB group may be due to an additive effect of the buckling to the inferior RD, supporting the inadequate effect of the intraocular tamponade for inferior RD.

In a previous published study, Quiram et al. [25] reported that in cases of retinectomy, a phacoemulsification with IOL implant is mandatory in order to perform anterior vitreous base dissection. In their study, both silicone oil tamponade and radical anterior base dissection with lens removal were associated with significantly better anatomical outcomes and this is in line with our results. In contrast, in their report authors did not find any significant improvement in anatomical success when scleral buckle was associated to an inferior retinotomy. However, retinectomy was not standardized at 180 degrees but ranged from a few clock hours to 360° with low repeatability of the results along with different surgeons carrying out the procedures. Additionally in their cohort, a mean of 1.8 previous surgical procedures (range 1 to 5) were performed, compared to our study population where all eyes underwent a single previous RD surgical repair.

Adelman et al. [47] in the first report of “European Vitreo-Retinal Society Retinal Detachment Study”, stated that a scleral buckle can offer a better anatomic results compared to PPV in phakic eyes with uncomplicated RD. The current study highlighted how this can be noted even for complicated RD where the SB can offer good support to the inferior sclera with better anatomical results. In the second “European Vitreo-Retinal Society Retinal Detachment” [48] authors analysed results for complicated RD surgery (with grade C PVR). They concluded that relaxing retinotomy could lead to significantly higher failure rates. However, the same authors specify that their findings about retinotomy should be interpreted with caution. Our results can therefore contribute as new data to revaluate the use of relaxing retinotomy, especially in cases of recurrent RD after primary SB procedure. In addition, our results are consistent with data published by Tseng et al. [34] demonstrating that when silicone oil is used as a postoperative tamponade in eyes with extensive inferior pathology, the placement of an inferior retinotomy may improve anatomical results. However our study is limited by its retrospective nature and the relatively small number of subjects in each subgroup. The small sample size of our study makes it difficult to establish evidence, but we speculate that our results can help clinicians consider scleral buckle surgery (when possible) in cases of primary inferior RD for phakic eyes in an era where the PPV approach has become more popular. Furthermore, we acknowledge that our results about visual acuity should be interpreted with caution. We cannot definitively attribute the improvement of visual acuity only to retinal status as it is difficult to affirm that we performed a clear lens extraction rather then a cataract surgery. In addition, despite the fact that, no statistically significant difference between the two groups were found in terms of retinal status, macula detachment was reported in 4 eyes out of 12 in the SB group and in 14 out of 21 in the PPV group. This data can explain the overall improvement in visual acuity at baseline and post-operatively in the SB group. Nevertheless, the study has several strengths, including the single surgeon performing the surgery, the long follow-up interval, the homogeneous subset of RDs and lens status.

## Conclusion

The present study re-enforces the value of relaxing retinotomy incisions in recurrent RD with grade C PVR, and does not support the view that relaxing retinotomies contribute to unfavourable anatomical outcomes in vitrectomy when performed by an expert surgeon. On the contrary, this study suggests that aggressive surgical management, in the setting of inferior retinal redetachment with complete traction relief thorough retinotomy, results in a high rate of successful reattachment, especially in eyes with pre-existing SB supporting inferior retinal breaks and receiving a silicone oil tamponade.

## Abbreviations

PVR: proliferative vitreoretinopathy
SB: scleral buckling
PPV: pars plana vitrectomy
RD: retinal detachment
VA: visual acuity
logMAR: logarithm of the minimum angle of resolution
BCVA: Best-corrected visual acuity
WHO: World Health Organization
IOL: intraocular lens
SD: standard deviation
